# Brain Stem and Entire Spinal Leptomeningeal Dissemination of Supratentorial Glioblastoma Multiforme in a Patient during Postoperative Radiochemotherapy: Case Report and Review of the Literatures

**DOI:** 10.1097/MD.0000000000000962

**Published:** 2015-06-19

**Authors:** Xiangyi Kong, Yu Wang, Shuai Liu, Keyin Chen, Qiangyi Zhou, Chengrui Yan, Huayu He, Jun Gao, Jian Guan, Yi Yang, Yongning Li, Bing Xing, Renzhi Wang, Wenbin Ma

**Affiliations:** From the Department of Neurosurgery, Peking Union Medical College Hospital, Chinese Academy of Medical Sciences, No. 1 Shuaifuyuan Hutong of Dongcheng District, Beijing, PR China.

## Abstract

Glioblastoma multiforme (GBM) is the most common primary malignancy of the central nervous system in adults. Macroscopically evident and symptomatic spinal metastases occur rarely. Autopsy series suggest that approximately 25% of patients with intracranial GBM have evidence of spinal subarachnoid seeding, although the exact incidence is not known as postmortem examination of the spine is not routinely performed.^1–3^ Herein, we present a rare case of symptomatic brain stem and entire spinal dissemination of GBM in a 36-year-old patient during postoperative adjuvant radiochemotherapy with temozolomide and cisplatin. Visual deterioration, intractable stomachache, and limb paralysis were the main clinical features. The results of cytological and immunohistochemical tests on the cerebrospinal fluid cells were highly suggestive of spinal leptomeningeal dissemination. After 1 month, the patient's overall condition deteriorated and succumbed to his disease. To the best of our knowledge, this is the first reported case of GBM dissemination presenting in this manner. Because GBM extracranial dissemination is rare, we also reviewed pertinent literature regarding this uncommon entity.

Although metastases to spinal cord from GBM are uncommon, it is always important to have in mind when patients with a history of GBM present with symptoms that do not correlate with the primary disease pattern.

## CASE REPORT

**FIGURE 1:**
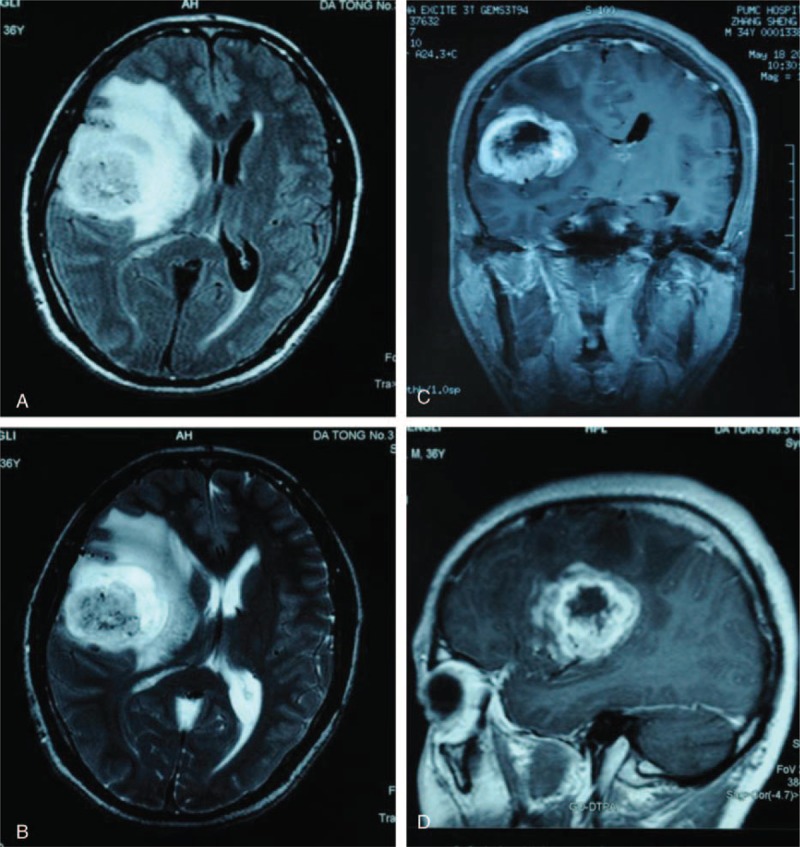
Radiological evaluation of the brain before surgery. T1-weighted axial gadolinium-enhanced magnetic resonance image demonstrates an enhancing tumor of the right frontal lobe (A, C, D). T2-weighted image demonstrates the same lesion as in the previous image, with notable tissue edema (B). This finding is consistent with a high-grade glioblastoma.

**FIGURE 2:**
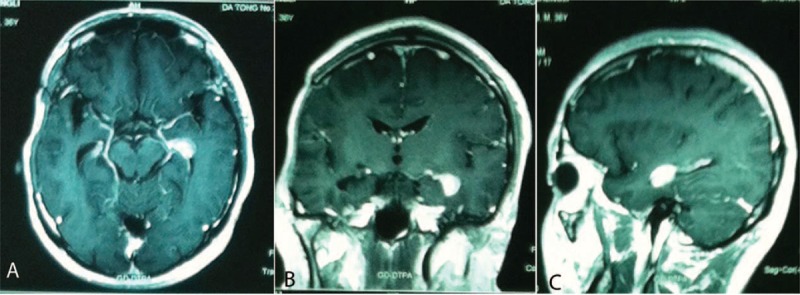
Radiological evaluation of the brain 4 months after surgery. T1-weighted axial gadolinium-enhanced magnetic resonance image demonstrates a new enhancing nearly semicircular lesion in the area of amygdala, uncus, and hippocampus.

A 36-year-old Chinese man, without special past medical history, complained of dull and persistent headache for 2 months. He also had involuntary salvation sometimes. He denied unconsciousness, convulsion, epilepsy or cognitive disorder, and noticed a slight weight loss despite a normal appetite. No special circumstances regarding his family history or personal history related to his presentation was identified. Neurological examination showed tendon hyperreflexia and normal function of sensation and movement. Pupils are round, equal in size, and constrict visibly to light. Visual acuity and visual field was normal. Pathological signs were absent. He had no problems with urination or defecation. Head magnetic resonance imaging (MRI) revealed a long-T1 and long-T2 mass measuring approximately 5 × 5 × 5 cm with obvious peritumoral edema in the right frontotemporal lobe. After injection of an intravenous contrast agent, the mass was heterogeneously ring-enhanced (Figure 1). The patient underwent a left temporal craniotomy and gross total tumor resection. The cut surface has a solid, pink appearance and the vascular supply was extremely rich. Immunohistochemistry staining showed glioblastoma multiforme (GBM) with E3 ubiquitin-protein ligase MIB (MIB)-1 (Ki-67) proliferation index of 23 to 25%, trioctyl phosphine oxide (TOPO) (++), and phosphatase and tensin homolog deleted on chromosome ten (PTEN) (++). No IDH1/IDH2 (isocitrate dehydrogenase [IDH]) mutation or methylguanine methyl transferase (MGMT) promoter hypermethylation were detected. The postoperative period was uneventful. Standard adjuvant radiotherapy (60 Gy in 30 fractions) and concurrent chemotherapy (temozolomide 75 mg/m^2^ per day) were performed. However, after 3 cycles of temozolomide (150–200 mg/m^2^ for 5 days during each 28-day cycle), a repeat MRI showed a new lesion in the area of amygdala, uncus, and hippocampus, which we highly suspected to be a metastasis (Figure 2). Considering the molecular pattern,^4^ an intravenous infusion of cisplatin (30 mg/m^2^ for continuous 3 days to a total of 100 mg during each 28-day cycle) was added together with temozolamide for 5 cycles.

**FIGURE 3:**
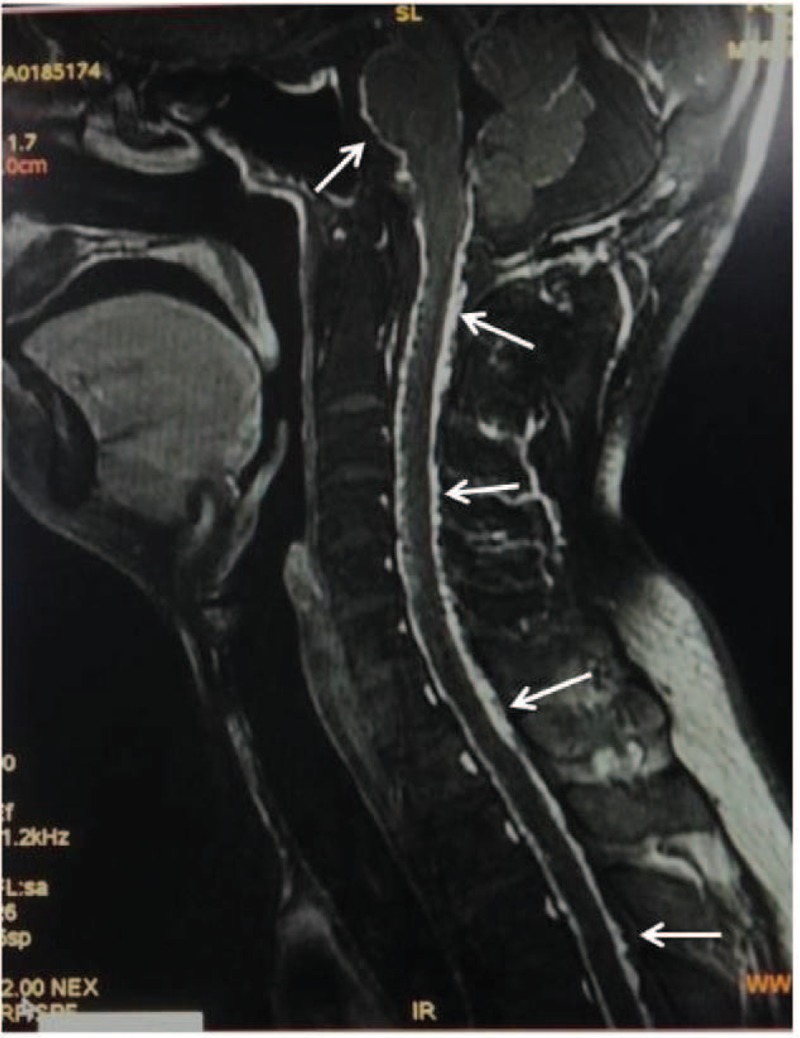
Radiological evaluation of the spinal cord. MRI T1 Gad sagittal image demonstrated the diffuse leptomeningeal enhancement (arrows). MRI = magnetic resonance imaging.

Two months later, the patient began to complain of numbness and weakness in his lower extremities, visual acuity's rapid decrease, serious weight loss, stomachache, and diarrhea. He was admitted to the ward and received symptomatic treatment, but the illness worsened precipitously. Only 2 days later, he could not move his legs and could only see things within 5 cm. The stomachache could not be alleviated by drugs and the diarrhea was more than 10 times each day. Physical examination showed a decreased pinprick sensation below S1 level. The muscle strength was 0/5 in right lower extremity, 1/5 in left lower extremity, bilateral upper limbs’ proximal strength was 3/5, and distal strength was 4/5. Muscle tone decreased and tendon reflexes weakened or disappeared. Bilateral Lasegue signs (+), nuchal rigidity (+), digital rectal examination (−), and stool routine and abdomen ultrasound detected no abnormality. A follow-up brain MRI showed no significant changes, but MRI of the spine showed widespread leptomeningeal tumor dissemination within the whole spinal cord and brain stem (Figure 3). Lumbar puncture showed an increased intracranial pressure more than 330 mmH_2_O. The cerebrospinal fluid (CSF) was beige and turbid. Total cell count in CSF was 13,523 × 10^6^/L, white blood cell was 3315 × 10^6^/L, protein content was 5.17 g/L, Cl^−^ 108 mmol/L, and glucose 2.4 mmol/L. Cytology was positive for sheets of malignant cells with pleomorphic nuclei with low ratio of nucleus to cytoplasm consistent with glial origin. Molecular and immunohistochemical tests showed glial fibrillary acidic protein (GFAP), PTEN, S-100 calcium-binding protein (S-100), human epidermal growth factor receptor (HER)4, and interleukin (IL)-13 are all positive. This constellation of findings was consistent with leptomeningeal seeding of GBM. The patient's family did not want further treatment and an autopsy was not done. The patient's overall condition deteriorated and succumbed to his disease 1 month later.

## DISCUSSION

GBM is the most aggressive type of gliomas, accounting for 12% to 15% of all intracranial tumors.^5^ First reported in 1931,^3^ CSF dissemination of GBM occurs in approximately 15% to 25% of GBM patients, although the accurate incidence reported in different studies ranged a lot.^6^ For infratentorial GBM, the incidence was higher. Witoonpanich once reviewed 600 GBM patients in a retrospective study, and found that symptomatic CSF dissemination just occurred in 2% of them.^7^ Intramedullary spinal metastases are much rarer.^8^ These results suggest that the symptomatic involvement occurs relatively late in the course of GBM. With the development of improved diagnostic tools, the incidence of CSF dissemination of GBM presents a gradual upward trend in the last few years. And with improvements in surgery, chemotherapy, and radiotherapy, prolonged survival time is observed, although the outcomes are still fatal.^6^

To date, it has been remained unclear which factors cause the metastasis of GBM.^2,9^ Cellular spread in the subarachnoidal space seems to be the most likely cause for spinal dissemination. In our case, the total cell count in CSF was extremely high while the white blood cell accounted for only a small part. CSF cytology was positive for sheets of malignant cells, which strongly suggested CSF dissemination. According to literature, ventricular entry at operation, repeated tumor resection, male sex, ependymal invasion, fissuring of the ependyma due to hydrocephalus, depressed immune function after radiotherapy and chemotherapy, and fragmentation of the tumor in contact with CSF were all associated with a statistically significant increased incidence of CSF dissemination.^10^ A pure biopsy for GBM tends to lead a shorter time to spinal metastasis development than tumorectomy.^11^

A meta-analysis showed there was no initial site predilection for supratentorial GBM that would make patients more prone to spinal metastases; and all cerebral lobes were equally involved. Contact with the CSF system was not a prerequisite.^12^ In our case, although the initial tumor was not adjacent to intracerebral CSF pathways, the widespread dissemination is obvious, which is in agreement with earlier findings. Lower thoracic, upper lumbar, and lumbosacral regions are the most common sites for spinal metastases of GBM. Nerve roots, cauda equina, nerve root sleeves, and the fundus of the thecal sac are also common sites of spread.^9,10^ The unique features of the present case lie in that the dissemination was so extensive and aggressive that the leptomeninges of the entire spinal cord and brain stem were invaded. Further, an intraspinal mass with abnormal signals was found at L5-S1 level on MRI indicating a possible intramedullary metastasis despite no biopsy.

Spinal metastases and CSF dissemination should be generally suspected in all GBM patients presenting with clinical manifestations that are not easy to be explained by the primary lesion.^9^ Backache and radiculopathy are the most common clinical manifestations of spinal leptomeningeal metastases, which is frequently followed by paraparesis and quadriparesis.^10^ In our case, the patient presented with rapid decrease of muscle strength and visual acuity, intractable stomachache, and frequent diarrhea, which were much different from previous reports. It is not easy to make an accurate clinical diagnosis of these conditions, but careful neurological examination directed at radicular signs and advanced medical imaging technologies especially spinal MRI with contrast enhancement are sure to help a lot.^13^

The prognosis of GBM patients with spinal dissemination is bleak and almost always leads to a fatal outcome. The mean survival time between diagnosis of dissemination and death is approximately 2 to 3 months.^14,15^ The treatment is chiefly palliative. Radiotherapy is the most common treatment of choice, with a total dose of 25–40 Gy, which may provide pain relief and some improvements of neurological function, but no survival benefits.^16^ Intravenous or intrathecal chemotherapy has not found to be very useful to improve the overall survival. Surgery may be attempted if there is a symptomatic, large metastatic deposit causing cord compression, but usually leptomeningeal dissemination is not amenable to surgery due to the diffuse nature of the disease.^16^ In our case, there was diffuse involvement of the spine, hence surgery was not possible.

One limitation of this case report is the lack of a tissue diagnosis of the drop metastasis. Although without a tissue diagnosis a second malignancy could not be ruled out, the results of cytological and immunohistochemical tests on the CSF cells were highly suggestive of spinal leptomeningeal dissemination.

## CONCLUSION

We report an uncommon case of brain stem and the whole spinal leptomeningeal dissemination of supratentorial GBM in a patient during postoperative adjuvant radiochemotherapy. With this paper, we emphasized the importance to suspect spinal metastases in all patients with a history of intracranial GBM who complain about symptoms or signs that cannot be explained by the primary lesion in spite of the rarity of this condition, because as treatment improvements provide better control of the primary tumor and improving survival, metastasis may occur.
